# Neatness

**Published:** 1872-03

**Authors:** 


### NEATNESS,

In its essence, and purely for its own sake, is found in few. Many a man is neat for appearance' sake; there is an instinctive feeling that there is power in it. When a man consults a physician or a lawyer for the first time, or comes to rent a house, or borrow money, he will come in his best dress; a lady will call in her carriage. A man who means business and honesty comes as he is, just as you will find him in his store, his shop, his counting-house. The most accomplished gamblers dress well; the most enterprising swindlers are faultlessly appareled; but countless multitudes are but whitewashed sepulchres. Too many "don't care, as long as it will not be seen." Washington Allston, the great artist, the accomplished gentleman, suddenly left his friend standing at the door of a splendid Boston mansion as they were about entering for a party, because he had just remembered he had a hole in his stocking. It could not be seen or known, but the very knowledge of its existence made him feel that he was less a man than he ought to be; it gave him a feeling of inferiority.

In any hundred met on Broadway and on the Avenue, a large percentage will be found, on the sudden removal of the stocking, to have dirt visible on the feet. A faultless glove too often covers black finger-nails; and the physician often finds inner garments to be in rags, or to have spots and stains too foul to think of.

As persons are less careless of personal cleanliness and tidy apparel, they are infallibly and necessarily less of the angel, more of the animal; more under the domination of passion, less under the influence of principle. Said a poor servant girl, "I can't explain what change religion has made in me, but I look more closely under the door-mat, when I sweep, than I used to." Intelligence, culture, elevation, give purity of body as well as purity of sense and sentiment.

Where you see a neat, tidy, cleanly, cheerful dwelling, there you will find a joyous, loving, happy family. But if filth, and squalor, and a disregard for the refining delicacies of life prevail in any household, there will be found in the moral character of the inmates much that is low, degrading, unprincipled, vicious, and disgusting. Therefore, as we grow in years, we ought to watch eagerly against neglect of cleanliness in person, and tidiness in dress.

